# DNA from *Lactobacillus paragasseri* SBT2055 Activates Plasmacytoid Dendritic Cells and Induces IFN-α via TLR9

**DOI:** 10.3390/microorganisms13071440

**Published:** 2025-06-20

**Authors:** Eiji Kobatake, Toshinobu Arai

**Affiliations:** Milk Science Research Institute, MEGMILK SNOW BRAND Co., Ltd., Saitama 350-1165, Japan

**Keywords:** plasmacytoid dendritic cells, type I interferon, interferon-α, lactic acid bacteria, *Lactobacillus paragasseri* SBT2055, Toll-like receptor 9

## Abstract

Previously, we reported that *Lactobacillus paragasseri* SBT2055 (LG2055) activates plasmacytoid dendritic cells (pDCs) and induces interferon alpha (IFN-α) in vitro. Our clinical trial suggested that LG2055 intake may enhance pDC activity, supporting immune maintenance and reducing subjective common cold symptoms. However, the precise mechanisms remain unclear. In this study, we investigated how LG2055 engages with pDCs to stimulate IFN-α production. We evaluated LG2055-induced pDC activation using flow cytometry, ELISA, and phagocytosis assays. Human peripheral blood mononuclear cells (PBMCs) were stimulated with LG2055 and its components to evaluate immune responses. An in vitro M cell model was used to examine LG2055 translocation. We found that DNA extracted from LG2055 activated pDCs and enhanced IFN-α production via Toll-like receptor 9 (TLR9). Phagocytosis assays demonstrated that LG2055 DNA was internalized by PBMC-derived pDCs, enabling TLR9-mediated signaling. Additionally, LG2055 translocated across M cells in vitro, suggesting potential transport into Peyer’s patches, where it may interact with pDCs. These findings demonstrate that intestinal LG2055 can translocate across M cells, interact with pDCs, and exert immune-stimulatory effects to enhance host antiviral immunity. This study provides mechanistic insight into how dietary components support immune health and could inform the development of novel functional foods or therapeutic strategies.

## 1. Introduction

After the global SARS-CoV-2 pandemic, individuals have placed greater importance on managing their physical health. Maintaining a well-functioning immune system is essential for protection against infectious diseases. Therefore, in recent years, growing interest has focused on enhancing immune function through the ingestion of beneficial ingredients in dietary habits.

*Lactobacillus paragasseri* SBT2055 (LG2055), formerly known as *Lactobacillus gasseri* SBT2055, is a strain of lactic acid bacteria (LAB) originally isolated from the intestine of a healthy adult [1,2]. Its safety for human consumption has been well established [3], and it has been used in dairy product manufacturing for over two decades. We have extensively investigated the health benefits of LG2055, particularly its immune-stimulating properties. Notably, LG2055 has demonstrated antiviral effects and the ability to promote immunoglobulin A (IgA) production in vivo [4,5,6]. Furthermore, our clinical study found that LG2055 intake increased hemagglutination inhibition titers against the influenza virus following vaccination while enhancing natural killer cell activity, salivary secretory IgA production, total plasma IgA and IgG levels, and antiviral gene expression [7]. These findings suggest that LG2055 may contribute to immune system enhancement and overall health maintenance. In randomized, double-blind, placebo-controlled studies, we reported that the intake of LG2055 was associated with a reduction in subjective common cold symptoms, such as runny nose, sneezing, and sore throat [8,9]. Additionally, LG2055 was found to activate plasmacytoid dendritic cells (pDCs), which play a key role in antiviral immune responses [9]. This suggests that the activation of pDCs may contribute to the immunomodulatory effects of LG2055 and its potential to improve host immune function.

pDCs are activated in response to viral stimulation and produce large amounts of type I interferons (IFNs). Once activated, pDCs also upregulate the expression of MHC Class II molecules, such as HLA-DR, as well as co-stimulatory molecules, such as CD40, CD80, and CD86, stimulating immune cells, including natural killer cells, B cells, and T cells, thereby enhancing the overall immune response, including innate and acquired immunity, and protecting the host from viral infections [10,11]. Therefore, activating pDCs through alternative means, such as the ingestion of LAB, may be beneficial for maintaining and enhancing immune function and host defense.

Our previous in vitro study demonstrated that LG2055 activates pDCs and promotes the production of IFN-α, a type I IFN, in human peripheral blood mononuclear cells (PBMCs) [9]. pDCs are known to be the primary source of IFN-α in response to stimulation with cytosine-phosphate-guanine (CpG) oligodeoxynucleotides [12,13,14]; therefore, LG2055 is presumed to induce IFN-α production in PBMCs via pDC activation. Moreover, a clinical trial revealed that the intake of LG2055 induces pDC activation, particularly in individuals with weakened immune function [9]. In the same trial, a questionnaire-based survey assessing daily common cold symptoms showed that participants in the LG2055 group experienced significantly fewer days with subjective symptoms compared to the placebo group. These findings suggest that LG2055 intake may activate pDC, thereby supporting immune system maintenance and alleviating common cold symptoms. Based on these results, we hypothesized that LG2055 consumption could help maintain physical health by effectively activating pDCs.

To interpret the results of our previous clinical trial, it is essential to understand how LG2055 interacts with pDCs. The gut immune system is believed to play a key role in facilitating contact between LG2055 and pDCs; however, this mechanism has not yet been elucidated. Additionally, the precise mechanisms underlying pDC activation and IFN-α production induced by LG2055 remain unclear. pDCs are known to highly express Toll-like receptor (TLR) 7 and TLR9, which trigger type I IFN production in response to single-stranded RNA and CpG DNA, respectively [10]. DNA from LAB activates pDCs via TLR9 [15]. TLR9 is a pattern-recognition receptor localized in endosomes and recognizes DNA derived from bacteria and viruses. Unmethylated CpG motifs, a specific sequence in DNA where a cytosine nucleotide is directly followed by a guanine nucleotide linked by a phosphodiester bond, activate TLR9, triggering immune responses. Mammalian DNA has a low frequency of CpG motifs, and most are methylated. In contrast, bacterial and viral DNA often contain unmethylated CpG motifs, making TLR9 an effective sensor of microbial DNA [16,17]. Human pDCs can also recognize Gram-positive bacteria via TLR1/2 [18]. While LG2055 has been shown to enhance IgA production through TLR2 on DCs [6], its specific effects on pDCs remain unknown. It is important to clarify these unresolved aspects. Therefore, this study aimed to investigate the mechanisms by which LG2055 activates pDCs and promotes IFN-α production in vitro.

## 2. Materials and Methods

### 2.1. Preparation of LG2055

LG2055 was cultured, harvested, and lyophilized as previously reported [9]. The resulting LG2055 powder was resuspended in phosphate-buffered saline (PBS) and used for subsequent assays. The bacterial count in the suspension was measured using a Bacteria Counting Kit for flow cytometry (Thermo Fisher Scientific, Waltham, MA, USA), following the manufacturer’s protocol.

### 2.2. DNA Extraction

LG2055 was cultivated in de Man, Rogosa, and Sharpe (MRS) broth (BD, Franklin Lakes, NJ, USA) at 37 °C for 16 h, harvested by centrifugation, and washed with PBS. DNA was extracted from the cell pellet using the MORA-EXTRACT kit (Kyokuto Pharmaceutical Industrial Co., Tokyo, Japan), following the manufacturer’s protocol. The double-stranded DNA concentration was measured using a NanoDrop One (Thermo Fisher Scientific).

### 2.3. Lipoteichoic Acid (LTA) and Peptidoglycan (PGN) Preparation

The lyophilized LG2055 powder was resuspended in sterile distilled water and disrupted five times using a French press (Ohtake, Tokyo, Japan) at a pressure of 180 MPa. Undisrupted cells were removed via centrifugation at 500× *g* for 5 min at 4 °C. The resulting supernatant was further centrifuged at 12,000× *g* for 5 min at 4 °C to separate the supernatant and pellet, which were used for LTA and PGN preparation, respectively.

LTA was prepared according to a previously published method [19] with several modifications. The supernatant of disrupted cells was filtered and stirred with an equal volume of 1-butanol for 30 min at room temperature. After stirring, the lower aqueous phase was collected by centrifugation at 5900× *g* for 20 min at 20 °C. The aqueous phase was concentrated, lyophilized, redissolved with 15% 1-propanol/1 M sodium acetate buffer (pH 4.7), and filtered to remove any insoluble material. The filtered solution was applied to a HiTrap™ Octyl FF column (GE Healthcare, Little Chalfont, UK), and the bound materials were sequentially eluted with 15%, 25%, 35%, and 45% 1-propanol/1 M sodium acetate buffer (pH 4.7). Each eluate was collected separately. The elution fractions of the applied sample and 15% 1-propanol solution were combined and reapplied to the column equilibrated with 15% 1-propanol/1 M sodium acetate buffer (pH 4.7). The elution and collection steps were repeated in the same manner. The eluates from 25% and 35% 1-propanol were combined as the fraction containing LTA. This fraction was then concentrated, dialyzed against distilled water using Slide-A-Lyzer™ G3 Dialysis Cassettes (3.5 K MWCO; Thermo Fisher Scientific), and lyophilized to obtain purified LTA. The presence of LTA was confirmed by dot blotting.

For PGN preparation, the pellet of disrupted cells was washed three times with distilled water and then lyophilized. PGN was prepared as described in our previous report [20].

### 2.4. DNA Digestion

To digest DNA extracted from LG2055, 1 mg/mL DNase I (from bovine pancreas; Roche, Roswell, GA, USA) was mixed with 0.5 mg/mL DNA in PBS. The mix was incubated at 37 °C for 1 h and then heated at 75 °C for 10 min to deactivate DNase I. As a control, DNA without DNase I treatment was processed under the same conditions. DNA digestion was confirmed via agarose gel electrophoresis.

### 2.5. PGN Digestion

To digest PGN purified from LG2055, 100 U/mL mutanolysin (from *Streptococcus globisporus*; Sigma-Aldrich, St. Louis, MO, USA) was mixed with 1 mg/mL PGN in 50 mM phosphate buffer (pH 6.5) and incubated at 37 °C for 1 h. PGN digestion was assessed by decreased turbidity and the disappearance of precipitate. Mutanolysin was inactivated by heating at 100 °C for 5 min. PGN without mutanolysin and mutanolysin without PGN were processed under the same conditions for comparison.

### 2.6. PBMC Culture

Human PBMCs from healthy donors were obtained from Lonza (Basel, Switzerland). PBMCs were cultured in Roswell Park Memorial Institute (RPMI) 1640 medium (Thermo Fisher Scientific), supplemented with 10% fetal bovine serum (FBS; Thermo Fisher Scientific), 100 U/mL penicillin, and 100 μg/mL streptomycin (Thermo Fisher Scientific), hereafter referred to as complete RPMI.

PBMCs were seeded in multi-well plates and cultured for 24 h at 37 °C under 5% CO_2_ with test substances. The following were used: LG2055 (10 μg/mL; approximately equal to 1 × 10^6^ cells/mL), DNA (1 μg/mL), LTA (10 μg/mL), PGN (10 μg/mL), muramyl dipeptide (MDP; 10 mg/mL; Cayman Chemical, Ann Arbor, MI, USA), Pam_3_CSK_4_ (1 μg/mL; InvivoGen, San Diego, CA, USA), and FSL-1 (1 μg/mL; InvivoGen). When adding DNA complexed with the transfection reagent 1,2-dioleoyl-3-trimethylammonium-propane (DOTAP) (DNA/DOTAP), the DNA diluted in HEPES-buffered saline (HBS; 20 mM HEPES, 150 mM NaCl) was mixed with double the volume of DOTAP methyl sulfate (Polysciences, Warrington, PA, USA) in HBS and allowed to stand for 10 min at room temperature. For the TLR9 inhibition assay, PBMCs were pretreated with 0.5 μM E6446 dihydrochloride (Selleck Chemicals, Houston, TX, USA) for 1 h before adding the test substances to the cells. Each experiment was performed using PBMCs derived from a single donor.

### 2.7. pDC Isolation from PBMCs

pDCs were isolated from PBMCs using the EasySep™ Human Plasmacytoid DC Isolation Kit (STEMCELL Technologies, Vancouver, BC, Canada) according to the manufacturer’s instructions. The purity of the isolated pDC fraction was confirmed to be greater than 95% via flow cytometry (Supplementary Figure S1). Briefly, dead cells were stained with Fixable Viability Stain 780 (BD), and the cells were stained with FITC anti-human Lineage Cocktail (CD3, CD14, CD16, CD19, CD20, CD56) (Sony, Tokyo, Japan), BV421 mouse anti-human CD123, APC mouse anti-human CD86, PE-Cy™7 mouse anti-human CD11c, and PE mouse anti-human HLA-DR (BD) antibodies. Live cells that were Lineage−, HLA-DR+, CD123+, and CD11c− were defined as pDCs.

### 2.8. Flow Cytometric Analysis of pDC Activity

To assess pDC activation, cells treated with the test substances were harvested and washed with PBS. Flow cytometric analysis was then performed as previously reported [9]. The definition of pDCs was the same as described above. The expression levels (mean fluorescence intensity) of cell surface markers (CD86 and HLA-DR) were measured and used as indicators of pDC activity.

### 2.9. Enzyme-Linked Immunosorbent Assay (ELISA)

The culture supernatants of PBMCs treated with the test substances were collected via centrifugation, and the concentrations of IFN-α and IFN-β were measured. The ELISA Flex: Human IFN-α (HRP) kit (MABTECH, Nacka Strand, Sweden) and the ELISA MAX™ Deluxe Human IFN-β kit (BioLegend, San Diego, CA, USA) were used according to the manufacturer’s instructions for ELISA.

### 2.10. Phagocytosis Assays

The lyophilized LG2055 powder was suspended in carbonate–bicarbonate buffer (Sigma-Aldrich) and stained with FITC-I (DOJINDO, Kumamoto, Japan) for 1 h at 37 °C. The stained cells were washed twice with PBS and then resuspended in PBS, resulting in FITC-labeled LG2055.

The isolated pDCs were seeded in a round-bottomed 96-well plate and incubated with FITC-labeled LG2055 (10 μg/mL; approximately equal to 1 × 10^6^ cells/mL) for 18 h at 37 °C under 5% CO_2_. After incubation, the cells were harvested and washed with PBS.

For immunocytochemistry, the harvested cells were applied to a Shi-fix™ Coverslip (Everest Biotech, Oxfordshire, UK) and allowed to attach at room temperature for 30 min. Excess cells were removed by gently washing with PBS, and the attached cells were fixed with 4% paraformaldehyde phosphate buffer solution (FUJIFILM Wako, Osaka, Japan) at room temperature for 20 min. The cells were washed three times with PBS, permeabilized with 0.1% Triton X-100/PBS for 10 min, and then washed three more times with PBS. Next, the coverslip was blocked with 1% bovine serum albumin (BSA)/PBS for 10 min. The cells were sequentially stained with the first antibody (HLA-DR Monoclonal Antibody (LN3), eBioscience™; Thermo Fisher Scientific) at a 1:1000 dilution in 1% BSA/PBS and the second antibody (Goat anti-Mouse IgG (H + L) Cross-Adsorbed Secondary Antibody, Alexa Fluor™ 594; Thermo Fisher Scientific) at a 1:1000 dilution in 1% BSA/PBS and then mounted on a slide using VECTASHIELD Vibrance Antifade Mounting Medium with DAPI (VECTOR LABORATORIES, Newark, CA, USA). Microscopic observation was performed using a BZ-X810 (KEYENCE, Osaka, Japan).

For flow cytometric analysis, the harvested cells were washed with Cell Staining Buffer (BioLegend) and fixed with Fixation Buffer (BioLegend) for 20 min at room temperature in the dark. The cells were washed again, resuspended in Cell Staining Buffer, and immediately analyzed. Fluorescence was measured using a FACSCanto II flow cytometer (BD), and the data were analyzed using FACSDiva software version 6.1.3 (BD). The cells incubated without LG2055 were used as the control, and the percentage of FITC-positive cells was calculated.

### 2.11. In Vitro M Cell Model

Caco-2 cells, a human colon carcinoma cell line, were purchased from the American Type Culture Collection (Manassas, VA, USA) and cultured in high-glucose Dulbecco’s Modified Eagle’s Medium (DMEM; FUJIFILM Wako) supplemented with 10% FBS, 1× non-essential amino acids (Thermo Fisher Scientific), 100 U/mL penicillin, and 100 μg/mL streptomycin, hereafter referred to as complete DMEM. Raji cells, a human Burkitt’s lymphoma cell line, were purchased from the European Collection of Authenticated Cell Cultures (Salisbury, UK) and cultured in complete RPMI. Both cell lines were cultured at 37 °C under 5% CO_2_.

Caco-2 and Raji cells were cocultured according to a previous report [21]. Caco-2 cells were seeded onto Transwell membranes (3.0 μm pore size) and cultured for 14 d, with the medium changed every 2–3 d. After 14 d, Raji cells suspended in a 1:2 mixture of complete RPMI:complete DMEM were added to the basolateral chamber of the Caco-2 monolayers. Coculturing was maintained for 6 d to induce M cell-like differentiation in Caco-2 cells. The medium on the apical side was changed every 2–3 d, and the resulting Caco-2 cells were used in assays as an in vitro M cell model. Caco-2 cells cultured alone under the same conditions served as the control.

After coculturing, the Transwells were transferred to a new plate, and the cells were washed twice with Hank’s balanced salt solution without calcium or magnesium (HBSS(-)) and without phenol red (FUJIFILM Wako), supplemented with 2% FBS and 25 mM HEPES (Thermo Fisher Scientific), hereafter referred to as complete HBSS. Complete HBSS was added to both the apical and basolateral sides of the Caco-2 monolayer and incubated at either 37 °C or 4 °C for 30 min. After equilibration, the Transwells were transferred to another plate containing complete HBSS, and the cells were washed again with complete HBSS. Subsequently, LG2055 (10^8^ colony-forming units/well) suspended in complete HBSS was added to the apical side, and the plate was incubated at either 37 °C or 4 °C for 150 min. After incubation, the complete HBSS from the basolateral side was collected for immediate measurement of viable bacterial counts. The collected supernatants were diluted 10-fold in complete HBSS and spread on MRS agar plates. The plates were incubated anaerobically at 37 °C for 3 d; the number of colonies formed was counted. Each experiment was performed in triplicate (*n* = 3).

### 2.12. Statistical Analysis

Data are presented as the mean ± standard deviation. Differences between groups were evaluated using one-way analysis of variance (ANOVA) followed by Tukey–Kramer or Dunnett’s *post hoc* tests for multiple comparisons. Student’s *t*-test was used for single comparisons. All statistical analyses were performed using EZR version 1.67 (Jichi Medical University, Tochigi, Japan) [22]. A *p*-value < 0.05 was considered statistically significant.

## 3. Results

### 3.1. LG2055 Induced pDC Activation and Promoted Type I IFN Production

**Figure 1:**
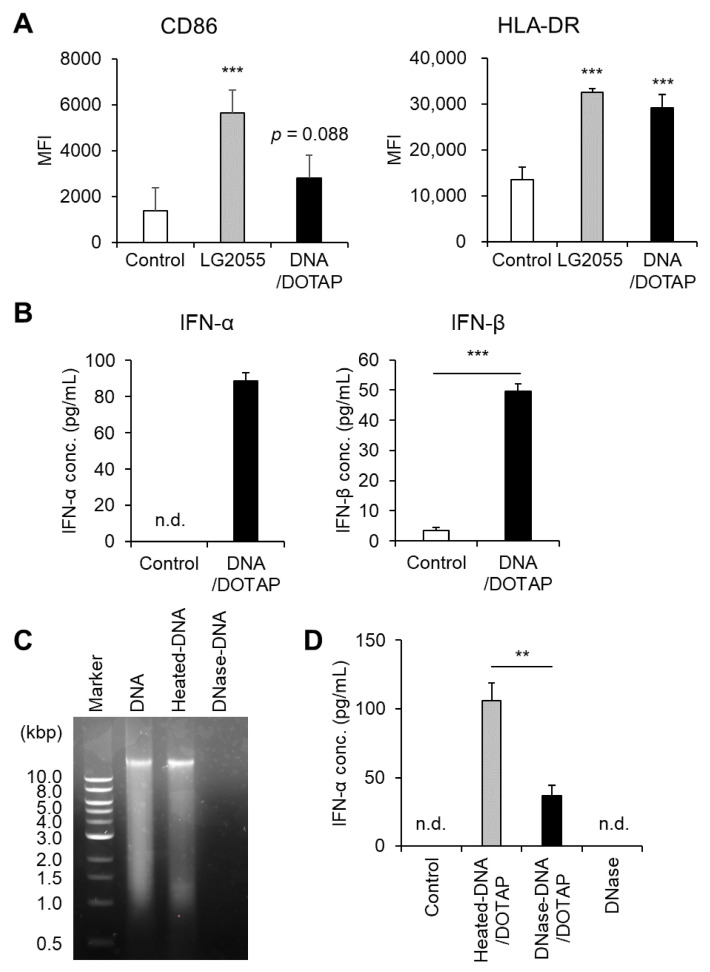
Plasmacytoid dendritic cell (pDC) activation and enhanced type I interferon (IFN) production by *Lactobacillus paragasseri* SBT2055 (LG2055) DNA: (**A**,**B**) Peripheral blood mononuclear cells (PBMCs) were treated with LG2055 or DNA/DOTAP for 24 h. (**A**) CD86 and HLA-DR expression on pDCs was evaluated using flow cytometry. (**B**) Concentration of type I IFNs (IFN-α and IFN-β) in the supernatant was measured via enzyme-linked immunosorbent assay (ELISA). (**C**) Agarose gel electrophoresis profile of DNase I-treated or non-treated LG2055 DNA. (**D**) PBMCs were exposed to DNase I-treated DNA/DOTAP, heat-inactivated DNA/DOTAP, or DNase I alone (no DNA) for 24 h. The concentration of IFN-α in the supernatant was measured using ELISA. (**A**,**B**,**D**) Each experiment was performed in triplicate (*n* = 3). Data are expressed as mean ± standard deviation. Test substances were added at a concentration of 10 μg/mL (LG2055) or 1 μg/mL (DNA). (**A**) *** *p* < 0.001 according to one-way analysis of variance (ANOVA) and Dunnett’s *post hoc* test (vs. Control). (**B**,**D**) ** *p* < 0.01 and *** *p* < 0.001 according to Student’s *t*-test.

First, we prepared bacterial components, including DNA, LTA, and PGN, from LG2055 cells and evaluated their ability to activate pDCs and enhance type I IFN production. LG2055 DNA complexed with the transfection reagent DOTAP significantly increased HLA-DR expression. Additionally, CD86 expression showed an increasing trend following DNA/DOTAP treatment (*p* = 0.088) (Figure 1A). Under these conditions, LG2055 LTA or PGN did not induce noticeable pDC activation (Supplementary Figure S2). Treatment with DNA/DOTAP significantly increased type I IFN (IFN-α and IFN-β) levels in PBMC culture supernatants (Figure 1B). To confirm the role of LG2055 DNA in type I IFN production, we digested it with DNase I and evaluated changes in IFN-α-promoting activity. DNase I treatment effectively degraded LG2055 DNA, whereas heat-treated DNA remained intact (Figure 1C). Moreover, DNase I-digested DNA exhibited a significant reduction in IFN-α-promoting activity compared to heat-treated DNA. DNase I alone (without DNA) did not induce IFN-α production (Figure 1D).

Activation of pDCs is associated with upregulation of cell surface markers such as CD86 and HLA-DR, as well as increased type I IFN production. Therefore, these results demonstrate that LG2055 DNA is a key activator of pDCs and a potent inducer of type I IFN production.

### 3.2. TLR9 Mediated LG2055 DNA-Induced Type I IFN Production

**Figure 2:**
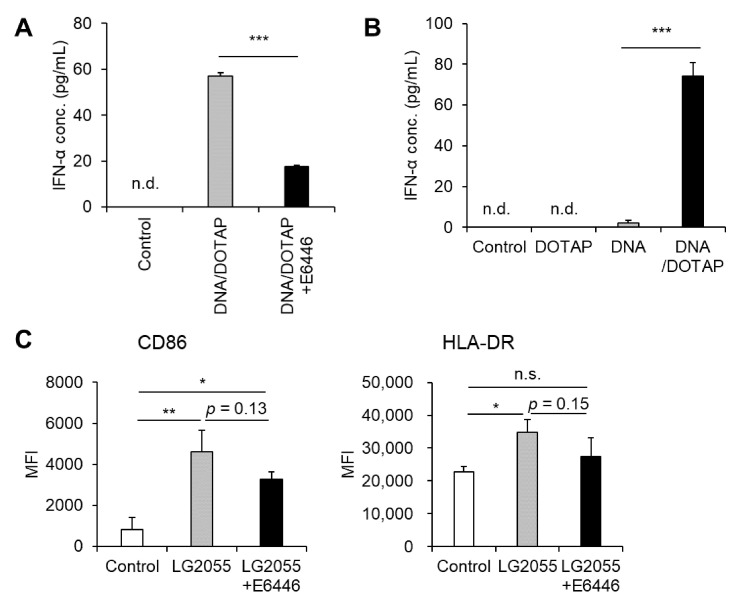
Toll-like receptor 9 (TLR9) is responsible for LG2055 DNA-induced IFN-α production: (**A**) PBMCs were pretreated with 0.5 μM E6446, a TLR9 inhibitor, for 1 h and then treated with DNA/DOTAP for 24 h. (**B**) PBMCs were treated with LG2055 and/or DOTAP for 24 h. (**A**,**B**) IFN-α concentration in the supernatant was measured using ELISA. (**C**) PBMCs were pretreated with 0.5 μM E6446 for 1 h and then treated with LG2055 for 24 h. CD86 and HLA-DR expression on pDCs was evaluated via flow cytometry. (**A**–**C**) Each experiment was performed in triplicate (*n* = 3). Data are expressed as mean ± standard deviation. Test substances were added at a concentration of 10 μg/mL (LG2055) or 1 μg/mL (DNA). (**A**,**B**) *** *p* < 0.001 according to Student’s *t*-test. (**C**) * *p* < 0.05 and ** *p* < 0.01 according to one-way ANOVA and Tukey–Kramer *post hoc* test.

TLR9 is a well-characterized receptor that recognizes bacterial DNA, triggering pDC activation and robust type I IFN production, including IFN-α [16,23]. Based on this, we hypothesized that LG2055 DNA enhances type I IFN production via TLR9. Indeed, pretreatment with the TLR9 inhibitor E6446 significantly reduced LG2055 DNA-induced IFN-α production (Figure 2A). Given that TLR9 is localized in intracellular endosomes, LG2055 DNA must be internalized by pDCs to be recognized by TLR9. Our data revealed that LG2055 DNA, when not complexed with DOTAP, did not significantly increase IFN-α production, whereas DNA/DOTAP promoted IFN-α production (Figure 2B). This suggests that DNA internalization is crucial for LG2055 DNA-induced IFN-α production. These findings indicate that LG2055 DNA is internalized by pDCs, recognized by TLR9, and contributes to increased type I IFN production.

Although LG2055 DNA is known to activate pDCs and induce type I IFN, its role in LG2055 cell-induced pDC activation remains unclear. Therefore, we examined whether LG2055 DNA contributes to LG2055 cell-induced pDC activation by blocking TLR9. Pretreatment with E6446 led to a slight reduction in the expression of pDC activation markers, CD86 and HLA-DR, in LG2055-treated pDCs (Figure 2C). This suggests that TLR9, which recognizes bacterial DNA, plays a role in LG2055 cell-induced pDC activation. Taken together, these results indicate that LG2055 DNA is at least partially responsible for LG2055-induced pDC activation via TLR9.

### 3.3. LG2055 Was Taken up by Intestinal M Cells and Phagocytosed by pDCs

**Figure 3:**
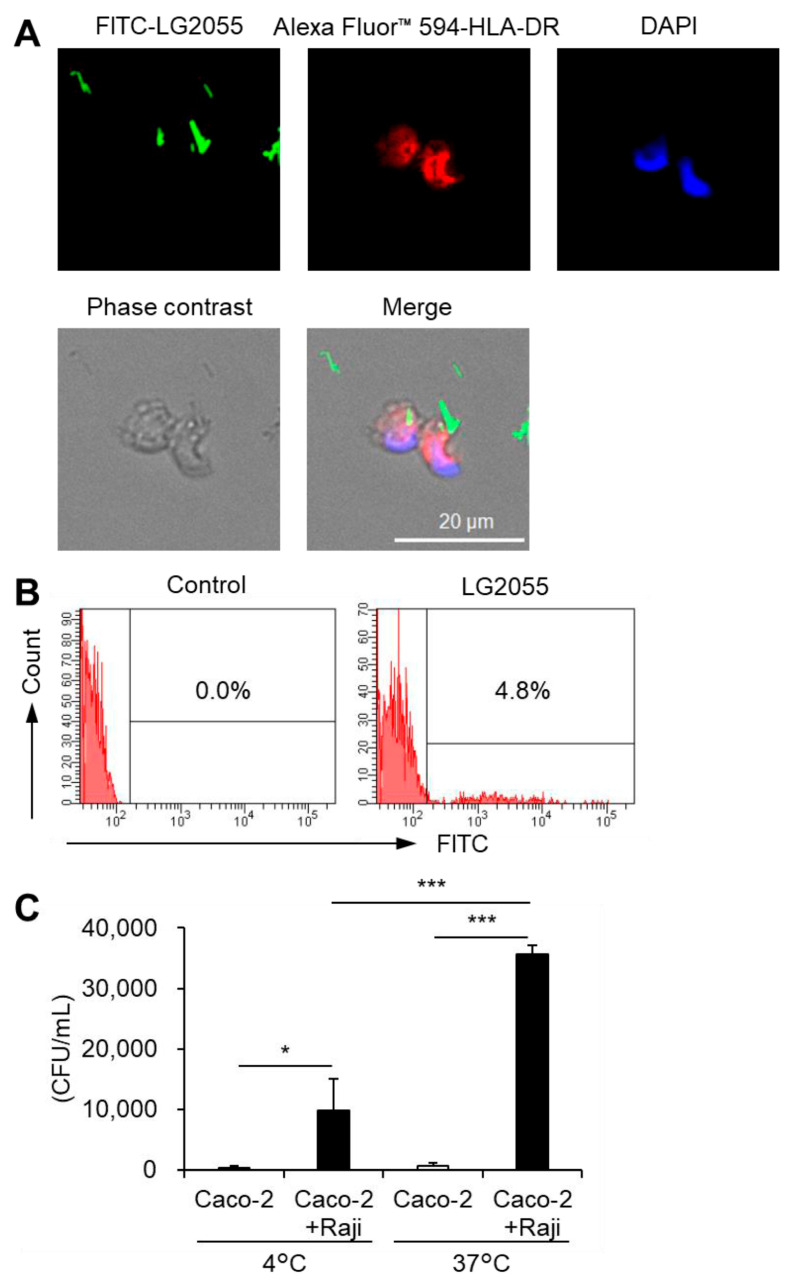
Phagocytosis of LG2055 by pDCs: (**A**,**B**) pDCs were isolated from PBMCs and treated with FITC-labeled LG2055 (10 μg/mL) for 18 h. The internalization of LG2055 was confirmed (**A**) by immunocytochemistry (scale bar: 20 µm) and (**B**) via flow cytometry. (**C**) LG2055 was transported across a Caco-2 monolayer at 37 °C or 4 °C. Transported LG2055 was quantified as colony-forming units (CFUs/mL). Each experiment was performed in triplicate (*n* = 3). Data are expressed as mean ± standard deviation. * *p* < 0.05 and *** *p* < 0.001 according to one-way ANOVA and Tukey–Kramer *post hoc* test.

As noted earlier, DNA internalization is crucial for promoting IFN-α production via TLR9. When LG2055 is administered as whole bacterial cells, it must be phagocytosed by pDCs to introduce its DNA intracellularly. To confirm this, we examined whether pDCs phagocytose LG2055 cells. Fluorescence microscopy of pDCs cocultured with FITC-labeled LG2055 cells confirmed LG2055 internalization by pDCs (Figure 3A). Moreover, flow cytometric analysis of FITC-labeled LG2055-treated pDCs detected a population of FITC-positive pDCs (Figure 3B), confirming LG2055 internalization by pDCs. These results demonstrate that pDCs can phagocytose LG2055 cells.

To interact with pDCs in the gut, LG2055 must first be transported into Peyer’s patches (PPs) via M cells. These specialized cells for the uptake of intestinal antigens are located in the follicle-associated epithelium of PPs. They play a crucial role in mucosal immunity. Therefore, we evaluated the translocation of LG2055 using an in vitro M cell model [21]. LG2055 was transported from the apical to basolateral side of M cells, and this transport was significantly reduced at a lower temperature (4 °C) (Figure 3C) compared to that at a higher temperature (37 °C). The in vitro M cell model has been reported to maintain barrier function at both 4 °C and 37 °C [21], suggesting that intestinal LG2055 is actively translocated across M cells to the basolateral side and that the cells detected on the basolateral side are not the result of passive diffusion. Taken together, these findings indicate that LG2055 is taken up by intestinal M cells, encounters pDCs in the PPs, and undergoes phagocytosis.

### 3.4. LG2055 PGN Suppressed IFN-α Production Induced by LG2055 DNA

**Figure 4:**
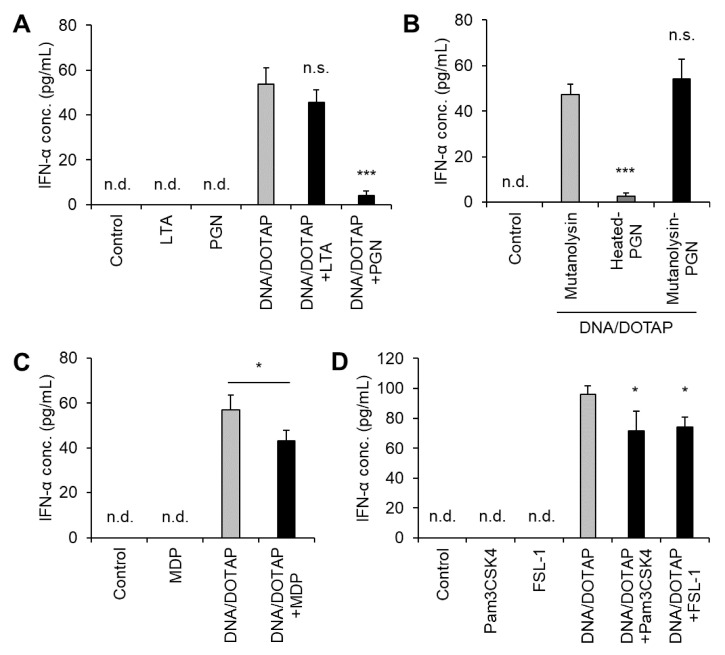
Suppression of LG2055 DNA-induced IFN-α production by peptidoglycan (PGN): PBMCs were treated with (**A**) DNA/DOTAP with or without bacterial components, lipoteichoic acid (LTA), or PGN; (**B**) DNA/DOTAP with or without mutanolysin-treated PGN; (**C**) DNA/DOTAP with or without muramyl dipeptide (MDP); or (**D**) DNA/DOTAP with or without Pam_3_CSK_4_ or FSL-1. (**A**–**D**) IFN-α concentration in the supernatant was measured using ELISA. Each experiment was performed in triplicate (*n* = 3). Data are expressed as mean ± standard deviation. Test substances were added at a concentration of 1 μg/mL (DNA, Pam_3_CSK_4_, and FSL-1) or 10 μg/mL (LTA, PGN, and MDP). (**A**,**B**,**D**) * *p* < 0.05 and *** *p* < 0.001 according to one-way ANOVA and Dunnett’s *post hoc* test, (**A**,**D**) vs. DNA/DOTAP, and (**B**) vs. mutanolysin + DNA/DOTAP. (**C**) * *p* < 0.05 according to Student’s *t*-test.

Jounai et al. reported that DNA from several LAB strains induces IFN-α production in murine bone marrow-derived Flt-3L-induced DCs and that LTA suppresses DNA-induced IFN-α production [23]. LTA is a key component of LG2055. When LG2055 is phagocytosed by pDCs, its DNA promotes IFN-α production alongside other bacterial components, including LTA. Therefore, we examined how other bacterial components influence LG2055 DNA-induced IFN-α production. Co-administration of LG2055-derived LTA did not suppress IFN-α production induced by LG2055 DNA. In contrast, co-administration of LG2055 DNA and PGN significantly reduced IFN-α production (Figure 4A). Mutanolysin-digested PGN did not suppress LG2055 DNA-induced IFN-α production, indicating that intact PGN itself, not another component in the preparation, is responsible for this effect (Figure 4B). Furthermore, MDP, a key PGN constituent and NOD2 ligand (Figure 4C), as well as the TLR2 ligands (Pam_3_CSK_4_ and FSL-1), only minimally suppressed IFN-α production (Figure 4D). These findings suggest that NOD2 and TLR2 contribute to the suppression of LG2055 DNA-induced IFN-α production.

## 4. Discussion

In this study, we demonstrated that DNA extracted from LG2055 activated pDCs and promoted IFN-α production via TLR9 (Figure 1 and Figure 2A). Dalpke et al. reported that DOTAP facilitates efficient bacterial DNA entry into cells, thereby activating TLR9 signaling [16]. Indeed, LG2055 DNA complexed with DOTAP significantly increased IFN-α production from PBMCs, whereas treatment with LG2055 DNA alone resulted in minimal IFN-α production (Figure 2B). These results support the hypothesis that LG2055 DNA enhances IFN-α production by engaging TLR9 within intracellular endosomes.

As described above, unmethylated CpG motifs are well known to activate TLR9. In addition, Ohto et al. reported that DNA containing cytosine at the second position from the 5′ end (5′-xCx DNA) is also crucial for efficient TLR9 activation. It has been shown that CpG DNA and 5′-xCx DNA bind to TLR9 simultaneously and cooperatively promote its activation [17]. Furthermore, Shimosato et al. reported that non-CpG oligodeoxynucleotides derived from *L. gasseri* JCM 1131^T^ induce Th-1 type cytokines via TLR9 signaling [24]. Although the specific sequences responsible for TLR9 activation in LG2055 DNA have not yet been identified, it is likely that multiple sequences contribute to this process.

For LG2055 to exert its effects as bacterial cells, it must be internalized, and its DNA must be delivered into cells. Our results demonstrated that pDCs phagocytosed LG2055 bacterial cells (Figure 3A,B). Phagocytosed LG2055 cells are likely digested, releasing DNA that can be recognized by TLR9. Indeed, TLR9 inhibition suppressed pDC activation by LG2055 cells (Figure 2C), indicating that LG2055 DNA is internalized through phagocytosis and exerts its effect via TLR9 when administered as bacterial cells.

In contrast, although DNA derived from *Lactobacillus rhamnosus* ATCC 53103 promotes IFN-α production, no increase in type I IFN expression or production has been observed when *L. rhamnosus* ATCC 53103 cells are used in vitro [23,25]. Moreover, *L. rhamnosus* ATCC 53103 was not taken up by pDCs, suggesting that the internalization of LAB is essential for pDC activation and subsequent IFN-α production [23]. Several studies have shown that the efficiency of LAB uptake by phagocytic cells, such as DCs and macrophages, varies depending on the bacterial species and strain [26,27]. Given that phagocytosis is triggered by interactions between phagocytic cells and bacteria [28,29], differences in the affinity between the cell surface receptors and bacterial components likely contribute to variations in bacterial uptake efficiency.

In this study, we used E6446 as a TLR9 inhibitor. Although E6446 can also inhibit TLR7 at relatively high concentrations [30], the concentration used in this study (0.5 μM) is sufficiently low to minimize off-target effects such as the inhibition of TLR7. Nonetheless, this potential cross-reactivity should be kept in mind. Additionally, we confirmed the essential role of DNA in IFN-α production (Figure 1D), supporting the conclusion that LG2055 DNA is recognized by TLR9, leading to enhanced IFN-α production.

Our previous clinical trials have demonstrated that the daily intake of LG2055 suppressed subjective symptoms of the common cold in healthy adults [8,9]. This effect is likely mediated by the enhancement of the host immune system through pDC activation. As well as the production of type I IFNs, activated pDCs stimulate various immune cells and enhance the overall immune response [10]; thus, pDC activation by LG2055 possibly contributes to suppressing the infectious symptoms. Several LAB strains, including LG2055, have been reported to activate pDCs and subsequently improve immune function, leading to the suppression of subjective common cold symptoms. For instance, clinical trials have shown that ingestion of *Lactococcus lactis* subsp. *lactis* JCM 5805 induces pDC activation and alleviates common cold symptoms [31,32]. Similarly, *Lactobacillus paracasei* MCC1849 has been found to activate pDCs and suppress subjective common cold symptoms in healthy adults [33,34]. These findings suggest that certain LAB species or strains share the ability to activate pDCs and enhance immune function. For LAB to activate pDCs and exert subsequent immune-stimulatory effects in humans, they must physically interact with pDCs. This emphasizes the importance of both the presence of specific bacterial components that promote pDC activation and IFN-α production as well as their ability to reach sites where they can exert these effects.

In this regard, the translocation of LAB by intestinal M cells is considered a key factor for LAB to exert their immune-stimulating effects in humans. The PPs in the small intestine are specialized tissues where various immune cells congregate and play a crucial role in intestinal immunity. M cells within PPs function to capture antigens, such as intestinal bacteria, and transfer them to DCs located within the PPs. Given that pDCs are found in PPs [35,36] and the adjacent lamina propria [37], these regions likely serve as sites where intestinal LAB, such as LG2055, interact with pDCs to exert their immunomodulatory effects.

As demonstrated in this study, LG2055 translocation by M cells was observed using an in vitro M cell model (Figure 3C), suggesting that LG2055 is transported into the PPs. Lactobacilli primarily colonize the small intestine [38], and LG2055, in particular, has been reported to exhibit a high ability for intestinal colonization [39]. These factors suggest that LG2055 has a relatively high frequency of contact with PPs, which may facilitate its transport into PPs via M cells. Therefore, it is likely that intestinal LG2055 is transported into the PPs, where it induces pDC activation and enhances immune function in humans.

We also demonstrated that PGN from LG2055 suppressed the IFN-α production induced by LG2055 DNA (Figure 4A). Previous studies suggest that IFN-α production via TLR9 can be suppressed by TLR2 or NOD2 signaling [40,41,42]. Given that TLR2 and NOD2 recognize bacterial PGN [43], it is proposed that LG2055 PGN suppressed IFN-α production through TLR2 or NOD2. However, treatment with MDP (a NOD2 ligand), Pam_3_CSK_4_ (a TLR1/2 ligand), or FSL-1 (a TLR2/6 ligand) resulted in only slight suppression of IFN-α production induced by LG2055 DNA (Figure 4C,D). This suggests that multiple signaling pathways may need to act simultaneously to achieve significant suppression of IFN-α production.

In addition, our data revealed that LG2055 LTA did not significantly suppress DNA-induced IFN-α production (Figure 4A), whereas Jounai et al. previously reported that LTA did suppress [23]. Typical LTA consists of a polymer moiety, comprising repeating glycerophosphate units, and a glycolipid anchor moiety, which is often dihexosyldiacylglycerol. However, LTA structures are known to exhibit diversity among species and strains [44]. LTA plays a crucial role in the innate immune response to Gram-positive bacteria, and structural differences may lead to variation in its immunomodulatory activity [45]. Regarding the discrepancy between the previous report and our data, Jounai et al. used commercially available LTA [23], whereas we prepared LTA directly from LG2055. It has been reported that *L. gasseri* JCM 1131^T^ LTA contains a specific glycolipid anchor structure, tetrahexosylglycerol with two or three acyl groups, and that this structure is common among multiple strains of the former *L. gasseri* species, including *L. paragasseri* [46,47]. These findings suggest that LG2055 LTA also contains a similar characteristic structure and that structural differences account for the varying effects on host cells compared to the previous report.

**Figure 5:**
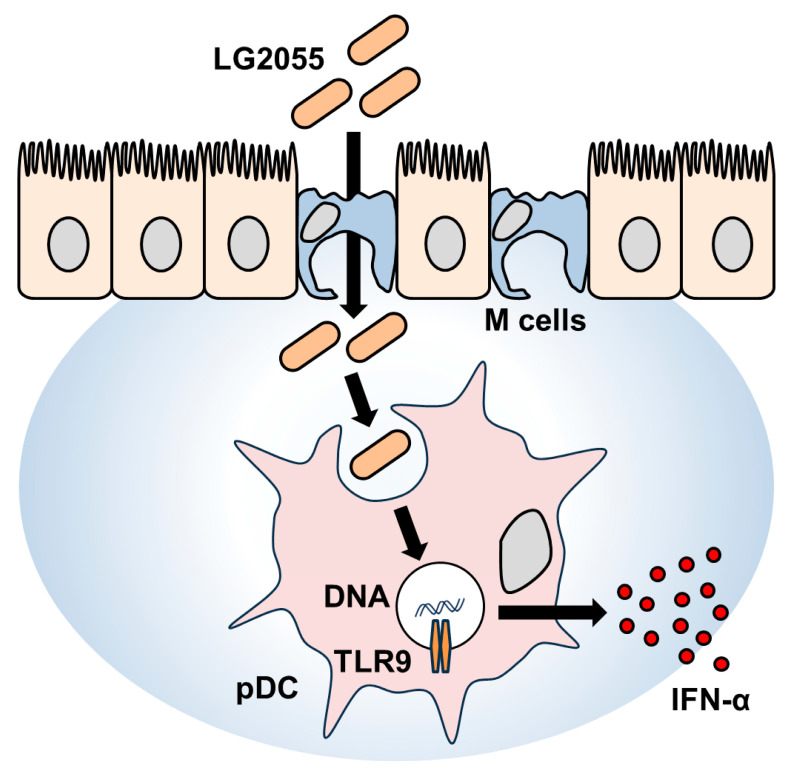
Schematic illustration of the mechanism underlying IFN-α production induced by LG2055.

In this study, LG2055 DNA demonstrated a significant promoting effect on IFN-α production. In contrast, our previous report has shown that LG2055 bacterial cells induced only a minimal increase in IFN-α production (<10 pg/mL) under the same experimental conditions [9]. Comparing these results, it can be inferred that in the case of bacterial cells, the combined action of LG2055 DNA and other bacterial components, such as PGN, may have contributed to the relatively lower levels of IFN-α production. Notably, the activation of pDCs and the induction of IFN-α production were clearly observed with LG2055 bacterial cells, indicating that the effects of LG2055 are effectively exerted in its bacterial form. Moreover, as mentioned above, LG2055 is likely translocated into PPs and subsequently phagocytosed by pDCs in its bacterial form in humans. Therefore, being in the form of bacterial cells is considered a crucial factor in maximizing the beneficial effects of LG2055 (Figure 5).

On the other hand, enhancement of the immune system can potentially trigger inflammatory responses. The relationship between inflammation and LAB has been well studied, with many reports indicating that LAB can help suppress inflammation [48,49]. LG2055, in particular, has also been shown to exert anti-inflammatory effects [50]. In addition, its antioxidative properties may contribute to suppressing inflammation by reducing reactive oxygen species [51]. Given that inflammation arises from various complex factors, it is important to consider both the beneficial and potentially adverse effects of immune activation by LG2055.

We demonstrated that LG2055 DNA contributes to the activation of pDCs and the promotion of IFN-α production through TLR9. However, we do not exclude the potential contributions of other bacterial components or signaling pathways. It is reasonable to consider that additional underlying mechanisms contribute to the effects of LG2055.

As noted previously, to interpret the results of our prior clinical study, we aimed to elucidate how LG2055 interacts with pDCs. In this study, we demonstrated the role of LG2055 DNA in pDC activation and IFN-α production via TLR9, as well as the translocation of LG2055 across M cells and its subsequent phagocytosis by pDCs. While similar phenomena have been reported for other LAB species or strains, comprehensive evaluations using specific LAB strains remain limited. It is well established that the biological functions of LAB vary across species and strains; therefore, compiling fundamental evaluations of individual strains is crucial to understanding their overall functionalities. Given that LG2055’s functionality has been demonstrated in several clinical trials, accumulating fundamental data on LG2055 is key to understanding its role within the broader LAB context.

The biological effects induced by LAB, including LG2055, are likely the result of a complex interplay between multiple bacterial components and/or metabolites interacting with the host. Variations in the structure or content of these components between species or strains may influence the overall balance of effects exhibited by each LAB, leading to differences in their activity. Future studies should focus on elucidating the intricate mechanisms underlying LAB-induced immune responses.

## 5. Conclusions

This study demonstrated that LG2055 is translocated by M cells, phagocytosed by pDCs, and induces pDC activation via TLR9, thereby promoting IFN-α production in vitro. These findings suggest that intestinal LG2055 can directly interact with pDCs and exert immune-stimulating effects, supporting the hypothesis that the intake of LG2055 may help maintain health by effectively activating pDCs. This insight may aid in the development of probiotic-based functional foods or supplements aimed at enhancing immune function.

## Data Availability

The raw data supporting the conclusions of this article will be made available by the authors on request.

## Supplementary Materials

The following supporting information can be downloaded at: https://www.mdpi.com/article/10.3390/microorganisms13071440/s1, Figure S1: Gating strategy of plasmacytoid dendritic cells (pDCs); Figure S2: LG2055 lipoteichoic acid (LTA) or peptidoglycan (PGN) did not induce noticeable pDC activation.

## Abbreviations

The following abbreviations are used in this manuscript:

ANOVA	Analysis of variance
BSA	Bovine serum albumin
CpG	cytosine-phosphate-guanine
DMEM	Dulbecco’s Modified Eagle’s Medium
DOTAP	1,2-dioleoyl-3-trimethylammonium-propane
ELISA	Enzyme-linked immunosorbent assay
FBS	Fetal bovine serum
HBS	HEPES-buffered saline
IgA	Immunoglobulin A
IFN	Interferon
LAB	Lactic acid bacteria
LG2055	*Lactobacillus paragasseri* SBT2055
LTA	Lipoteichoic acid
MDP	Muramyl dipeptide
MRS	de Man, Rogosa, and Sharpe
PBMC	Peripheral blood mononuclear cell
PBS	Phosphate-buffered saline
PGN	Peptidoglycan
pDC	Plasmacytoid dendritic cell
PPs	Peyer’s patches
RPMI	Rosswell Park Memorial Institute
TLR	Toll-like receptor
