# Supplementary material for: DNA from Lactobacillus paragasseri SBT2055 Activates Plasmacytoid Dendritic Cells and Induces IFN-α via TLR9
Source: Microorganisms. 2025 Jun 20;13(7):1440. doi: 10.3390/microorganisms13071440 (PMC12298028; doi:10.3390/microorganisms13071440)
Supplement: Supplementary file 1 [file microorganisms-13-01440-s001.zip › microorganisms-3670679-supplementary.pdf]

Supplementary materials

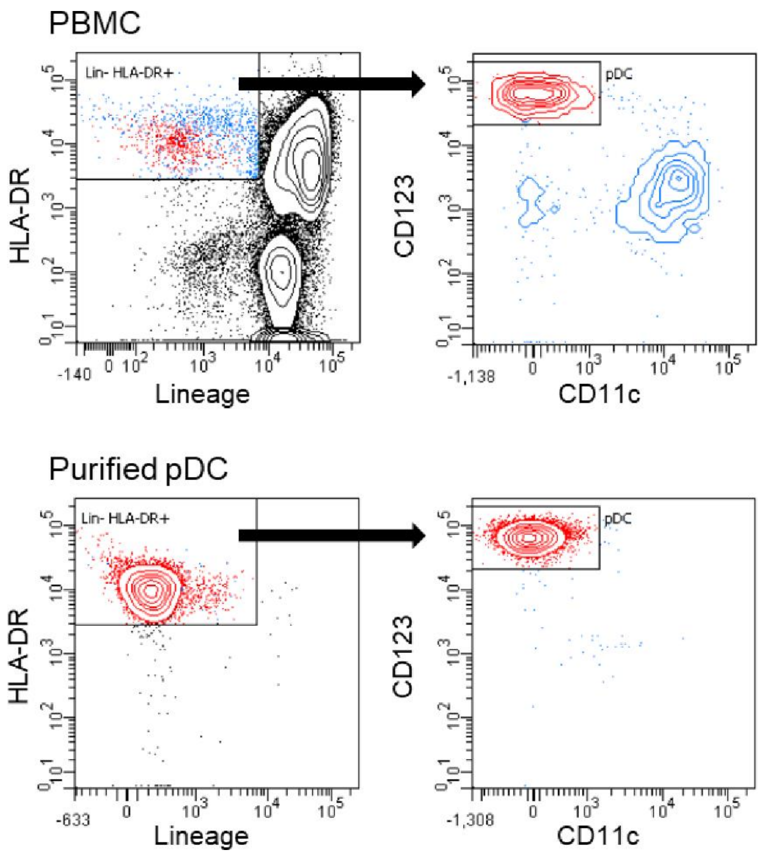

Supplementary Figure S1. Gating strategy of plasmacytoid dendritic cells (pDCs): Live cells that were Lineage−, HLA-DR+, CD123+, and CD11c− were defined as pDCs.

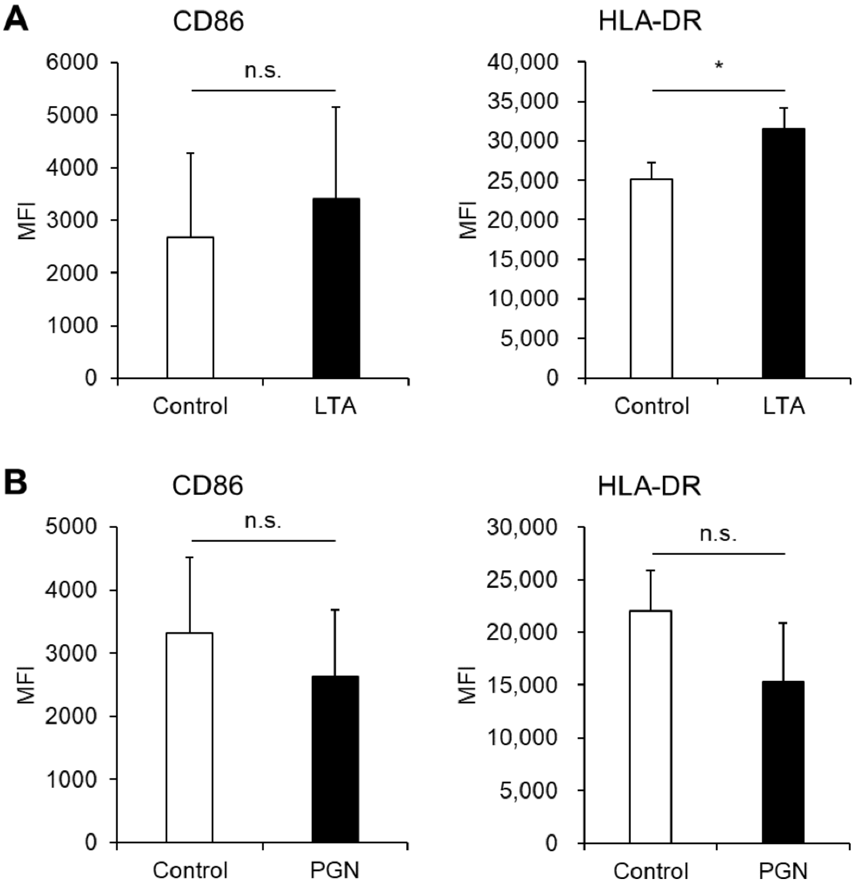

Supplementary Figure S2. LG2055 lipoteichoic acid (LTA) or peptidoglycan (PGN) did not induce noticeable pDC activation: Peripheral blood mononuclear cells (PBMCs) were treated with LG2055 LTA or PGN for 24 h. CD86 and HLA-DR expression on pDCs was evaluated using flow cytometry. Each experiment was performed in triplicate (n = 3). Data are expressed as mean ± standard deviation. Test substances were added at a concentration of 1 μg/mL. * $p < 0.05$ according to Student's t-test.
